# Survey of Leafhopper Species in Almond Orchards Infected with Almond Witches'-Broom Phytoplasma in Lebanon

**DOI:** 10.1673/031.011.6001

**Published:** 2011-05-09

**Authors:** Hala A. Dakhil, Efat Abou-Fakhr Hammad, Choaa El-Mohtar, Yusuf Abou-Jawdah

**Affiliations:** Department of Agricultural Sciences, American University of Beirut, Lebanon

**Keywords:** Aphrodinae, *Asymmetrasca decedens*, Cicadellidae, Deltocephalinae, Megophthalminae

## Abstract

Leafhoppers (Hemiptera: Auchenorrhyncha: Cicadellidae) account for more than 80% of all “Auchenorrhynchous” vectors that transmit phytoplasmas. The leafhopper populations in two almond witches'-broom phytoplasma (AlmWB) infected sites: Tanboureet (south of Lebanon) and Bourj El Yahoudieh (north of Lebanon) were surveyed using yellow sticky traps. The survey revealed that the most abundant species was *Asymmetrasca decedens*, which represented 82.4% of all the leafhoppers sampled. Potential phytoplasma vectors in members of the subfamilies Aphrodinae, Deltocephalinae, and Megophthalminae were present in very low numbers including: *Aphrodes makarovi*, *Cicadulina bipunctella*, *Euscelidius mundus*, *Fieberiella macchiae*, *Allygus theryi*, *Circulifer haematoceps*, *Neoaliturus transversalis*, and *Megophthalmus scabripennis*. *Allygus theryi* (Horváth) (Deltocephalinae) was reported for the first time in Lebanon. Nested PCR analysis and sequencing showed that *Asymmetrasca decedens*, *Empoasca decipiens*, *Fieberiella macchiae*, *Euscelidius mundus*, *Thamnottetix seclusis*, *Balclutha* sp., *Lylatina inexpectata*, *Allygus* sp., and *Annoplotettix danutae* were nine potential carriers of AlmWB phytoplasma. Although the detection of phytoplasmas in an insect does not prove a definite vector relationship, the technique is useful in narrowing the search for potential vectors. The importance of this information for management of AlmWB is discussed.

## Introduction

Almond witches'-broom phytoplasma (AlmWB), ‘*Candidatus* Phytoplasma phoenicium’, caused devastating damage to almond production in Lebanon, killing over 100,000 trees over the last decade. The symptoms included witches'-brooms arising mainly from the main trunk and roots, early flowering, stunted growth, dieback, off-season growth, and proliferation of slender shoots. The causal organism was identified as a phytoplasma closely related to, but distinct from members of the pigeon pea witches'broom phytoplasma group (16SrIX) (Abou-Jawdah et al. 2002; Verdin et al. 2003). AlmWB was also reported as a major almond disease in Iran (Verdin et al. 2003; Salehi et al. 2006), and more recently it was reported to be a real threat to peach and nectarine in South Lebanon (Abou-Jawdah et al. 2009).

The rapid spread of AlmWB over large geographical areas suggested the presence of an efficient vector. Most natural transmissions of phytoplasmas occur via phloem-feeding hemipteran insects, primarily leafhoppers (Cicadellidae, suborder “Auchenorrhyncha” Sorensen et al. 1995) (Boudon-Padieu et al. 1989). Within Cicadellidae, the largest number of vector genera and species occur in the subfamily Deltocephalinae, which also encompasses the greatest number of nonvector species of leafhoppers (Harris 1979).

Other known hemipteran vectors include planthoppers (Cixiidae), psyllids (Psyllidae), and froghoppers (Cercopidae) (Davies and Eyre 1996; Weber and Maixner 1998; Jarausch et al. 2001). Few phytoplasma vectors were reported in the suborder Heteroptera and they comprised three stinkbug species in the genus *Halyomorpha* (Hemiptera: Pentatomidae) (Hiruki 1998). In the Cicadellidae, male genitalia are the primary character to identify and classify nearly all species and most genera. Characters of similar value are almost completely lacking in females. The aedeagus is the most consistently used character in leafhopper species' differentiation (Ribaut 1936, 1952).

The development of polymerase chain reaction (PCR) assays recently provided a sensitive tool for phytoplasma detection (Davis et al. 1992; Jarausch et al. 1994). Nested PCR is now widely used for sensitive and reliable diagnosis of phytoplasmas in fruit trees (Lorenz et al. 1995). Although detection of phytoplasmas in an insect species does not prove a vector relationship, the technique is useful for narrowing the search for potential vectors among many taxa (Vega et al. 1993; Weintraub and Beanland 2006).

The objective of the present study was to survey leafhopper species in two AlmWB affected areas in Lebanon and to identify potential vectors of AlmWB phytoplasma using nested PCR technique and sequencing. It also reports for the first time the occurrence of *Allygus theryi* (Horvath) in Lebanon (Dakhil 2002).

## Materials and Methods

### Description of study sites

Two sites with high almond witches'-broom incidence were selected for the survey in 2001 and 2002: Tanboureet (Saida district, south of Lebanon) and Bourj El-Yahoudieh (Tripoli district, north of Lebanon). The Tanboureet (300 m altitude) site was primarily an established almond (*Prunus amygdalus* L.) orchard in which trees were 6–10 years old.

Some grapevines were present in the area. Weeds between the rows were removed regularly and intermittent insecticide sprays were applied during the survey period. The landscape surrounding this site was a mixture of lemon, loquat, grapevine, and olive. The orchard in Bourj-El Yahoudieh (190 m altitude) was composed primarily of 18 yearold almond trees. Some wild grapevines were present. The almond orchard was neglected and therefore weeds were not removed and no insecticides were sprayed during the survey period. The almond orchard was bordered by pine, grapevine, and olive trees. A complementary survey of leafhopper populations was conducted during late spring—early summer of 2004 in two almond orchards infected with witches'-broom phytoplasma (AlmWB), located at Bourj El-Yahoudieh (Tripoli district) and at Bsebeel (Zhgarta district) located north of Lebanon. Only leafhoppers from the latter survey were used for Phytoplasma detection.

### Insect Trapping

Yellow sticky traps were deployed at each of the surveyed locations. Six hand-made yellow sticky traps (22 × 15 cm) were deployed at each site, 1.5 m above ground level. Starting mid-November 2001 until end of May 2002, yellow sticky traps were replaced at biweekly intervals. A complementary survey using yellow sticky traps was conducted during the spring-early summer 2004. This survey was conducted for AlmWB phytoplasma PCR testing in trapped leafhoppers, which was not conducted during the 2001-02 survey.

### Leafhopper Identification

Insect collections were sorted and only leafhopper taxa were kept. Leafhoppers removed from the sticky yellow traps, were treated with toluene for 5 min followed by absolute ethanol for another 5 min. This procedure was performed in order to insure complete glue removal from leafhoppers. Specimens were preserved in 75% ethanol until identification.

Dark colored specimens were observed directly under the microscope after dissecting the aedeagus, whereas light colored specimens needed to be stained prior to microscopic observation as follows: leafhopper specimens were put in a few ml of 10% KOH, a drop of Chlorazol Black E (Merck, www.syngentacropprotection.com) was added, then the specimens were placed in an oven at 60° C for 1 hour (Carayon 1969).

For microscopic observations, a drop of glycerine was put on a slide on which dissection of the leafhopper's aedeagus was performed. Identifications were made to the genus or species level according to Ribaut (1936, 1952), Linnavuori (1962), Dlabola (1974), Ossiannilsson (1983), Delia Giustina (1989), and Abdul-Nour (1986, 1987a, 1987b, 1988, 2001).

**Table 1.**
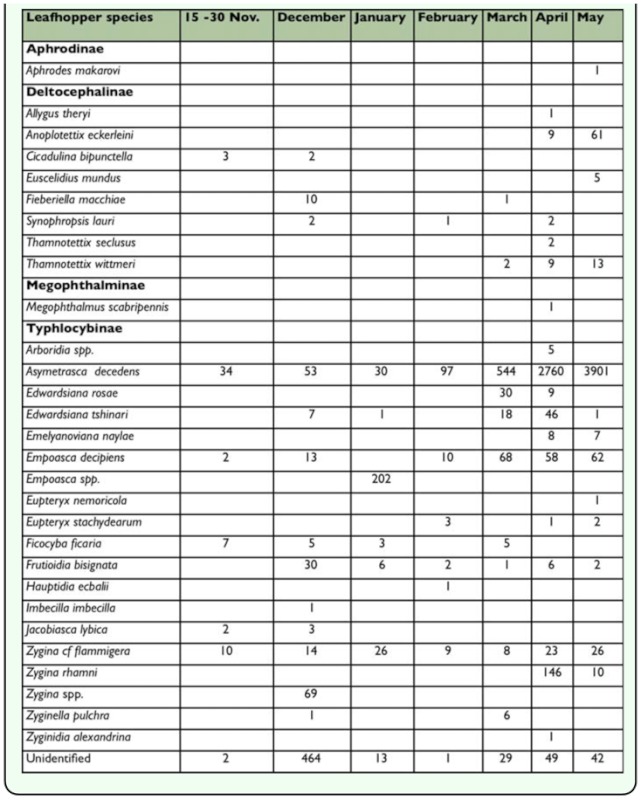
Leafhopper species collected in sticky yellow traps at Bourj El Yahoudieh (District Tripoli, North of Lebanon) during a period of 6.5 months (2001/2002)

**Table 2.**
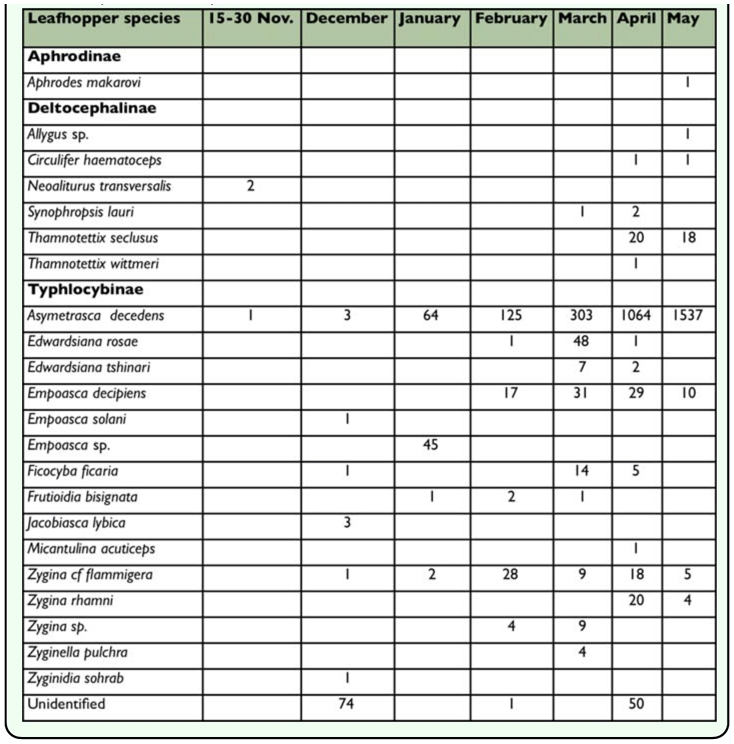
Leafhopper species collected in sticky yellow traps at Tanboureet (District Saida, South of Lebanon) during a period of 6.5 months (2001/2002)

### Nucleic Acid Extraction

Leafhoppers collected during the spring—early summer 2004 survey were identified to the genus and species level, then DNA from single leafhoppers [or five in the case of *Zygina* sp., *Empoasca decipiens* (Paoli), and *Asymmetrasca decedens* (Paoli)] was extracted by the small-scale nucleic acid protocol (Zhang et al. 1998) with minor modification to the extraction buffer [2% CTAB, 1.4 M NaCl, 20 mM EDTA, 1% polyvinylpyrolidone (PVP), 100 mM Tris-HCl pH = 8.0 and 0.2% mercaptoethanol (added just before use)]. Grinding of the insects was done on ice with a pestle attached to an electric drill. The pellet was washed in 75% ethanol, air dried, and suspended in 50µl TE buffer.

**Table 3.**
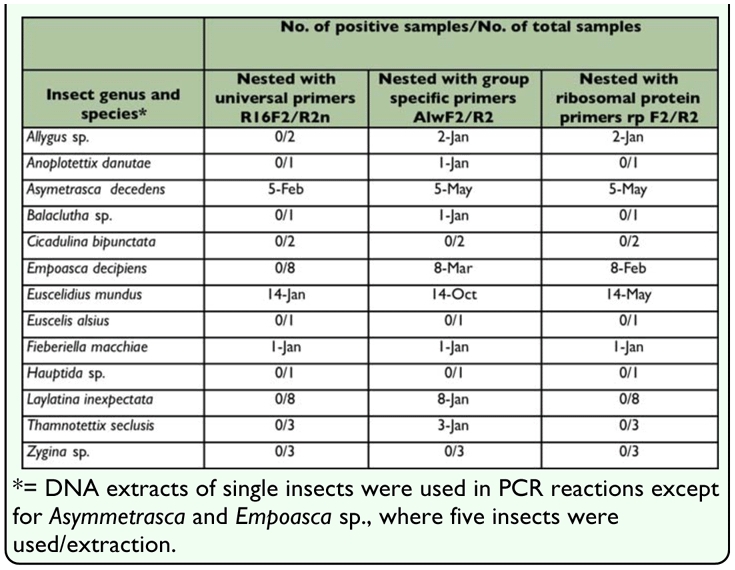
Detection of AlmWB in leafhoppers by three different nested PCR protocols (2004)

### PCR Amplification

Five primer pairs were used in this study: two pairs of universal primers, P1/P7 (Schneider et al. 1995) and R16F2n/R16R2 (Gundersen and Lee 1996); a group specific primer pair AlwF2/R2 (Abou-Jawdah et al. 2003); and two primer pairs for a gene encoding a ribosomal protein (rpF1/R1 and rpF2/R2). Nested PCR was performed in all cases. R16F2/R2 (Gundersen and Lee 1996) and Alw F2/R2 (Abou-Jawdah et al. 2003) were nested after a PCR run by the universal primer pair P1/P7 (Schneider et al. 1995). As for the ribosomal protein encoding gene primer pair Rp F2/R2, it was nested after a PCR run by another primer pair Rp F1/R1 (Lee I.M. personal communication). PCR amplifications were performed in 20µl reactions containing 20OmM of dNTPs, 0.2µM of each primer (forward and reverse), 2.5 mM MgCl_2_, 1x polymerase buffer, 1 unit Taq polymerase enzyme (AB gene), and 1.6µl DNA sample. PCR reactions were carried out in an Icycler (Bio-Rad, www.bio-rad.com) as follows: one cycle at 95° C for 2 min; 35 cycles (94° C for 30 sec, 50° C for 30 sec, 72° C for 2 min), and a final extension step at 72° C for 7 min. In the nested PCR run with the group specific primers, the program was modified whereby annealing was set at 46° C for 30 sec and extension at 72° C for 45 sec. The PCR products were separated by electrophoresis in 1.2% agarose gels and stained in ethidium bromide.

### Sequencing

The PCR products were cloned in pGEMT® easy vector (Promega, www.promega.com) and sequenced at the Molecular and Cellular Biology Core Facility, Faculty of Medicine, American University of Beirut using 3100 — Avant Genetic Analyzer (Applied Biosystems, www.appliedbiosystems.com). The resulting nucleotide sequence was compared to the published sequences using the BLASTN 2.2.3 program (Altschul et al. 1997).

## Results

### Survey of leafhoppers

During the sampling period extending from mid-November 2001 until the end of May 2002, a total of 12,756 leafhoppers were collected of which 12,029 were identified. Leafhoppers collected represented 27 genera belonging to 4 subfamilies. In the two agroecosystems surveyed (Bourj El-Yahoudieh and Tanboureet), the predominant subfamilies were Typhlocybinae (15 genera) followed by Deltocephalinae (9 genera). Each of the remaining subfamilies, Aphrodinae and Megophthalminae, were represented by only one species; *Aphrodes makarovi* (Zachvatkin) and *Megophthalmus scabripennis* Edwards, respectively (Tables 1 and 2).

In the subfamily Typhlocybinae, *Asymmetrasca decedens* (Paoli) was the most abundant species, representing 82.4% of all the leafhoppers sampled. In general, the population of *A. decedens* was higher in Bourj El Yahoudieh than in Tanboureet. *Empoasca decipiens* (Paoli) (2.35%) was the second most abundant species in this survey. The other species were: *Empoasca* spp. (1.94%), *Zygina rhamni* Ferrari (1.41%), *Zygina cf flammigera* (1.4%), *Edwardsiana rosae* Linnaeus (0.7%), *Edwardsiana tshinari* Zachvatkin (0.64%), *Zygina* spp. (0.64%), *Frutioidia bisignata* Mulsant et Rey (0.4%), and *Ficocyba ficaria* Horváth (0.31%) (Tables 1 and 2).

As for the subfamily Deltocephalinae; the most abundant leafhopper species were *Anoplotettix eckerleini* Dlabola and *Thamnotettix seclusus* Linnavuori (Table 1 and 2), which represented 0.54% and 0.31%, respectively, of all leafhoppers identified in this survey. Other deltocephalids were found in lower numbers including *Fieberiella macchiae* Linnavuori (Table 1), *Cicadulina bipunctella* (Matsumura) (Table 1), and *Neoaliturus transversalis* (Puton) (= *Circulifer transversalis*) which were captured in November and December (Table 2) while *Euscelidius mundus* (Haupt) (Table 1) and *Circulifer haematoceps* (Mulsant and Rey) (Table 2) were caught during April and May.

**Figure 1.**
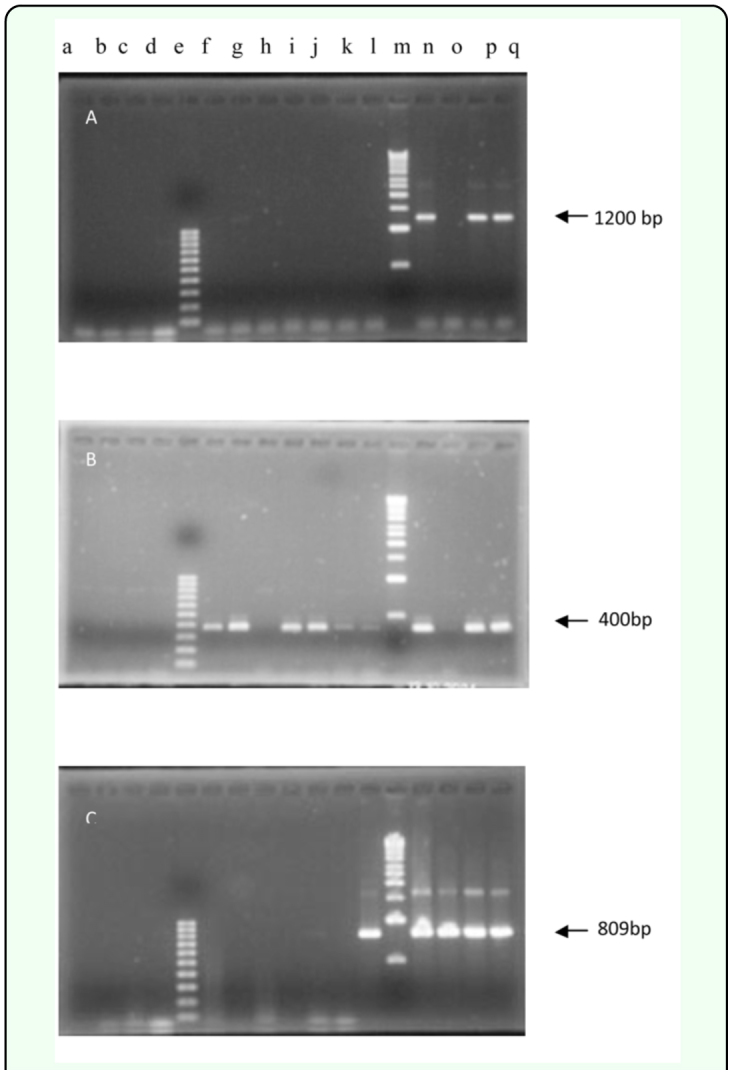
Agarose gel electrophoresis (1.2%) of nested PCR products obtained from DNA extracts of several leafhopper species. (A) Nested PCR with universal primer pair R16F2/R2n after PCR by universal primer pair P1/P7. (B) Nested PCR with group specific primer pair ALw F2/R2 after PCR by universal primer pair P1/P7. (C) Nested PCR with ribosomal protein primers F2/R2 after PCR by ribosomal protein primer pair F1/R1. a = *Zygina* sp.; b = *Cicadulina bipunctata*; c = Euscefe *alsices*; d = *Recilia chmidtgeni*; e & m = 100 and 1000 base pair ladders, respectively; f = *Thamnottetix sedusis*; g = *Balclutha* sp.; h & i = *Lylatina inexpectata*; j = *Allygus* sp.; k = *Annoplotettix danutae*; I = *Empoasca decipiens*; n & o = *Euscelidius mundus*; p = *Asymmetrasca decedens*; and q = *Fieberiella macchiae*. High quality figures are available online.

### AlmWB Detection in leafhoppers by PCR

During the complementary survey conducted, 12 genera of leafhoppers were trapped and identified (Table 3). Nested PCR analysis using universal primer pair P1/P7 followed by R16F2n/R16R2, showed that DNA extracts from *Asymmetrasca decedens*, *Euscelidius mundus*, and *Fieberiella macchiae* were positive for the presence of phytoplasma as determined by the characteristic 1200 bp amplified DNA fragments (Figure 1A; Table 3). Nested PCR with the group specific primers was more sensitive and detected phytoplasma in the three leafhopper species mentioned above and in six additional taxa: *Thamnottetix seclusis*, *Balclutha* sp., *Lylatina inexpectata*, *Allygus* sp.,__*Annoplotettix danutae*, and *Empoasca decipiens* (Figure 1B; Table 3).

Sequence comparison of the nested PCR products with the NCBI Genbank showed 99% identity (homology) with the AlmWB phytoplasma ribosomal RNA-encoding gene (Accession numbers: AF 455041, AF455040, AF 455039, AF 455038, AF 455038, AF 390137, AF 390136). Nested PCR with primers specific for ribosomal proteins confirmed the presence of phytoplasma in *Allygus* sp., *Asymmetrasca decedens*, *Empoasca decipiens*, *Euscelidius mundus*, and *Fieberiella macchiae* (Figure 1C; Table 3). Sequencing and multiple alignment of the nested PCR products from two of these leafhoppers showed that they all were most closely related to AlmWB phytoplasma with an identity of over 99%.

## Discussion

A major gap in the epidemiology of the reported AlmWB phytoplasma, which caused devastating losses to almond and nectarine crops in Lebanon, is caused by the fact that the vector is still unknown (Abou-Jawdah et al. 2002, 2003). Leafhoppers account for more than 80% of all “Auchenorrhynchous” phytoplasma vectors (Harris 1979). The present study focused on the identification of leafhoppers encountered in almond orchards between mid-November 2001 and end of May 2002. Preliminary screening was also conducted to identify leafhoppers that may carry phytoplasma in order to narrow the search for identification of potential AlmWB vectors in future studies, knowing that this does not exclude the possibility that the vector might belong to another family such as the Psyllidae (Carraro et al. 1998, 2001).

In this study, the highest number of leafhoppers trapped belonged to the subfamily Typhlocybinae. In this subfamily, a total number of 15 genera and 18 species were identified, of which *Asymmetrasca* followed by *Empoasca* were the most prevalent during this survey period. Starting in early March an increase in *Asymmetrasca decedens* population was noticed in the two surveyed sites: Bourj El Yahoudieh (north of Lebanon) and Tanboureet (south of Lebanon). The population of *A. decedens* was higher at Bourj El Yahoudieh than at Tanboureet; this might be due to differences in management practices and plant biodiversity. At Tanboureet the almond orchard was sprayed with pesticides and the weeds were controlled, while at Bourj El Yahoudieh the orchard was neglected and several weed species that could harbor leafhoppers were present. Most leafhoppers in the subfamily Typhlocybinae are mesophyll feeders (Nault and Rodriguez 1985); this fact reduces the possibility that they may act as potential phytoplasma transmitters. However, *Asymmetrasca decedens* and *Empoasca decipiens*, the two predominant species in this survey were found to be carriers of AlmWB by nested PCR. Similar results were reported for these two genera in Italy, where they were found positive for ESFY in nested PCR assays and transmission trials verified the ability of *E. decedens* to transmit ESFY from *Prunus armeniaca* L. to *P. armeniaca* (Pastore et al. 2004). More recently, out of 67 *Empoasca* spp. samples examined by PCR in Cuba, 63 were found carrying ‘*Candidatus* Phytoplasma aurantifolia’ (Arocha et al. 2006).

The subfamily Deltocephalinae was represented by a total of nine genera and 10 species. The most common genera were *Thamnotettix*, *Anoplotettix*, *Euscelidius*, and *Fieberiella*. Nested PCR assays revealed that *Fieberiella macchiae*, *Euscelidius mundus*, *Thamnottetix seclusis*, *Balclutha* sp., *Lylatina inexpectata*, *Allygus* sp., and *Annoplotettix danutae* were carriers of AlmWB. The subfamily Deltocephalinae includes about 60% of the vector genera and 59% of the vector species that transmit 70% of the known phytopathogenic agents (Nielson 1979). Some of the identified genera like *Euscelidius* (Caudwell et al. 1972; Palermo et al. 2001), *Fieberiella* (Jensen 1957; Purcell et al. 1987; Krezal et al. 1989), *Euscelis* (Marzachi et al. 1998; Alma et al. 2000), *Cicadulina* (Nielson 1985), *Circulifer* and *Neoaliturus* (Fos et al. 1986; Golino et al. 1987), and *Allygus* (Hegab et al. 1986) have been reported to transmit phytoplasma.

Furthermore, *Allygus theryi* (Horváth) was reported for the first time in Lebanon and was found to carry AlmWB phytoplasma by two out of three PCR protocols. It is worth mentioning that two *Allygus* spp. were reported to transmit the western-X-disease and peach yellows disease (Hegab et al. 1986).

The subfamily, Aphrodinae, was represented by *Aphrodes makarovi* (Zachvatkin); some *Aphrodes* species are capable of transmitting phytoplasmas (Nielson 1979). The Megophthalminae subfamily was represented by *Megophthalmus scabripennis* Edwards, which was reported to be positive by PCR for grapevine phytoplasma (Orenstein et al. 2003).

During the present study, nested PCR analysis showed that *Fieberiella macchiae*, *Euscelidius mundus*, *Asymmetrasca decedens*, *Thamnottetix seclusis*, *Balclutha* sp., *Lylatina inexpectata*, *Allygus* sp., *Annoplotettix danutae*, and *Empoasca decipiens* were nine potential phytoplasma carriers. Phytoplasma normally multiply in their vectors and become transmissible only after they accumulate to high levels in the posterior acinar cells of the salivary glands (Kirkpatrick 1992). Among the three nested PCR primers used, the group specific primer pair was the most sensitive while the universal primer pair was the least sensitive. Detection of phytoplasma by the least sensitive method in a single insect in *Euscelidius mundus* and *Fieberiella macchiae* may indicate that phytoplasma have accumulated to a high titer in the insect bodies and therefore present an indication for their possible role as AlmWB vectors. However, detection of AlmWB in the salivary glands supported by transmission tests will be required to confirm this observation. Another interesting observation is the detection of AlmWB phytoplasma, as confirmed by sequencing, in *Asymmetrasca decedens* in the three PCR protocols used and in the five composite samples assayed. This is in agreement with recent reports from Italy that detected European Stone Fruit Yellows (16 SRX-B) phytoplasma in *Empoasca decipiens*, which is a close relative to *Asymmetrasca decedens* (Pastore et al. 2004).

Some vectors often may be found colonizing the diseased host; while other vectors feed only occasionally on the diseased crop and normally colonize weeds or other plant species. This might explain why genera in the subfamilies Deltocephalinae, Aphrodinae, and Megophthalminae that include known vectors of phytoplasmas (Nielson 1979) were not trapped in large numbers and were not present throughout the survey period. Therefore, leafhoppers such as *Fieberiella* and *Euscelidius*, which were only occasionally present in almond orchards during the survey period, may be the vectors responsible for transmission between orchards. Even though *Asymmetrasca decedens* may not be an efficient vector, it is the major leafhopper present in almond and nectarine orchards and may play a role in spreading AlmWB phytoplasma within orchards. The possible role of other vectors should not be overlooked especially that the number of leafhoppers tested by PCR is considered low. The probability of the occurrence of more than one potential vector species may explain the rapid spread of this disease in Lebanon.

As the main potential vectors feed on weeds and are found only occasionally on almond trees, effective weed management in and around the orchard supplemented by pesticide sprays to control leafhoppers were quite effective in limiting the spread of the AlmWB in Tanboureet when they were combined with eradication of all infected almond trees as soon as symptoms appeared. At present, three years after eradication of all infected trees and their replacement by new almond seedlings, the new seedlings look quite healthy.

AlmWB may be considered a serious quarantine organism for many countries, and effective control measures should be undertaken. Eradication should be considered in order to limit the spread of the disease, identification of possible insect vector(s) and the alternative hosts are key elements on which successful control strategies should be based. This study showed that the rapid spread of the disease might be related to the presence of more than one potential vector. However, the mere detection of phytoplasma in an insect by PCR is not proof that the insect does transmit the phytoplasma (Vega et al. 1993; Weintraub and Beanland, 2006). Therefore, molecular tools should be complemented with biological assays to prove whether the leafhopper carriers of phytoplasma reported in this study do transmit the AlmWB phytoplasma to almond or other stone fruit trees, and to determine the efficiency and modality of transmission.
